# TNFAIP8 promotes the migration of clear cell renal cell carcinoma by regulating the EMT

**DOI:** 10.7150/jca.40191

**Published:** 2020-03-04

**Authors:** Mengya Zhong, Maoshu Zhu, Yu Liu, Ying Lin, Lianghai Wang, Yuhan Ye, Huiyu Chen, Yan Yang, Guohong Zhuang, Jiyi Huang

**Affiliations:** 1 Xiang'an Branch, The First Affiliated Hospital of Xiamen University, Xiamen, Fujian, China.; 2 Cancer Research Center, School of Medicine, Xiamen University, Xiamen, Fujian, China.; 3 The Fifth Hospital of Xiamen, Xiamen, Fujian, China.; 4 Department of Gastrointestinal Surgery, Zhongshan Hospital Affiliated to Xiamen University, Xiamen, Fujian, China.; 5 Department of Pathology, Shihezi University School of Medicine, Shihezi, Xinjiang, China; 6 Department of Pathology, Zhongshan Hospital Affiliated to Xiamen University, Xiamen, Fujian, China.; 7 Organ Transplantation Institute of Xiamen University, Fujian Provincial Key Laboratory of Organ and Tissue Regeneration, School of Medicine, Xiamen University, Xiamen, Fujian, China.

**Keywords:** TNFAIP8, EMT, ccRCC, migration, metastasis

## Abstract

**Background**: Clear cell renal cell carcinoma (ccRCC) is characterized by high metastatic potential, and the epithelial-mesenchymal transition (EMT) has been shown to play a key role in multiple cancer progression, migration and metastasis and is the leading cause of poor prognosis. Currently, tumor necrosis factor-α-induced protein 8 (TNFAIP8/TIPE) is a newly discovered tumorigenesis factor, and TNFAIP8 and the EMT influence the migration of renal cancer cells.

**Methods**: In this study, we first analyzed the relationship between TNFAIP8 and ccRCC using bioinformatics, followed by immunohistochemistry to evaluate the relationship between the two in clinical samples. Subsequently, reverse transcription PCR and western blotting confirmed the expression of TNFAIP8 in ccRCC cells. Furthermore, we measured the migration and invasion abilities by using wound healing and transwell assays after overexpression or knockdown of TNFAIP8 in cells. In addition, we verified whether TNFAIP8 affects the EMT process in ccRCC by quantitative real-time PCR, western blotting, immunohistochemistry and immunofluorescence experiments.

**Results**: Through database analysis, we found that TNFAIP8 was highly expressed in ccRCC patients and was positively correlated with tumor stage and grade, indicating that TNFAIP8 is associated with the development of advanced ccRCC and poor prognosis. We subsequently confirmed that TNFAIP8 was abnormally overexpressed in clinical samples and ccRCC cell lines and that TNFAIP8 promoted ccRCC cell migration and invasion in vitro. Finally, we found that TNFAIP8 regulated EMT-related molecule expression and regulated the EMT process.

**Conclusion**: High expression of TNFAIP8 reinforces migration and regulates the EMT in ccRCC, conferring the metastatic potential of ccRCC and suggesting that TNFAIP8 may be a potential therapeutic target for the treatment of advanced ccRCC.

## Introduction

Renal cell carcinoma (RCC) is the second most malignant tumor in the urinary system, accounting for 2% to 3% of adult malignant tumors 1, 2. Among them, clear cell renal cell carcinoma (ccRCC) is the most common form of RCC, and the proportion is approximately 85% 3. In addition to the increasing global incidence, the prognosis of ccRCC is extremely poor, mostly because patients have already discovered distant metastasis at the time of diagnosis, and patients with ccRCC metastases currently face limited clinical treatment 4, 5. Therefore, it is urgent to find new molecular biomarkers for the diagnosis of ccRCC at an early stage.

The TIPE (tumor necrosis factor-α induced protein 8) family are newly described regulators of immunity and tumorigenesis and are composed of four highly homologous proteins: TNFAIP8 (TIPE), TNFAIP8L1 (TIPE1), TNFAIP8L2 (TIPE2) and TNFAIP8L3 (TIPE3) 6, 7. TNFAIP8, also known as SCC-S2, GG2-1, NDED, and MDC-3.13, was the first described member of the TIPE family. It is upregulated and induced by NF-κB metastatic head and neck squamous cell carcinoma cell lines, and protects cancer cells from TNFα-induced apoptosis 8, 9. Although overexpression of TNFAIP8 in a variety of tumor cell lines enhances tumor proliferation and migration 10, it is unknown that TNFAIP8 exerts clinically meaningful effects and related mechanisms in RCC, particularly in ccRCC.

The epithelial-mesenchymal transition (EMT) is a reversible process in which epithelial cells lose polarity, adhesion and tight junctions and gain migratory and invasive abilities 11, 12. The EMT is involved in a variety of physiological processes, such as embryonic development, morphogenesis, and wound healing 13. In cancer, the EMT promotes tumor progression and invasion and produces blood resistance. At present, up to 30% of patients with RCC have distant metastasis, and the EMT of tumor cells is a potential process that drives tumor progression, invasion, and metastasis 14-16. To elucidate the pathological significance of the EMT in the occurrence, development, and metastasis of ccRCC, as well as to explore treatments based on targeted regulation of EMT-crucial molecules, is a key scientific issue in the study of EMT mechanisms in tumor metastasis.

Therefore, this study sought to determine the role of TNFAIP8 in ccRCC, whether it involves the EMT process, and whether it affects the expression of EMT marker molecules. Through biological information analysis tools and clinical sample testing, we found that TNFAIP8 is highly expressed in ccRCC, which is closely related to migration and metastasis. The biological effects of TNFAIP8 on migration and invasion were also studied. In addition, overexpresssion of TNFAIP8 downregulated EMT-specific epithelial genes (such as E-cadherin and ZO-1) and upregulated mesenchymal genes (such as N-cadherin and Vimentin). These results provide new insights for TNFAIP8 in the development of ccRCC and may be helpful for the treatment of advanced ccRCC.

## Materials and Methods

### ccRCC tissue collection

Samples from patients with ccRCC were collected from the Department of Pathology of Zhongshan Hospital of Xiamen University. After surgical removal, the tissues were immediately made into paraffin tissue blocks and tissue sections. In the absence of consideration for age, sex, ethnicity, or cancer stage, we randomly selected samples for subsequent experiments. All human samples were obtained with informed consent and approved by the ethics committee of Zhongshan Hospital of Xiamen University. The diagnosis was confirmed as ccRCC based on the World Health Organization (WHO) and International Society of Urological Pathology (ISUP) criteria. Therefore, the samples were also divided into 4 grades: grade 1 = tumors with nucleoli that are inconspicuous and basophilic at ×400 magnification; grade 2 = tumors with nucleoli that are clearly visible at ×400 magnification and eosinophilic; grade 3 = tumors with clearly visible nucleoli at ×100 magnification; and grade 4 = tumors have extreme pleomorphism or rhabdoid and/or sarcomatoid morphology. Tumors were staged based on the eighth edition of the tumor-node-metastasis (TNM) classification of the American Joint Committee on Cancer (AJCC): T1 = tumor ≤7 cm in the largest dimension, limited to the kidney; T2 = tumor >7 cm in the largest dimension, limited to the kidney; T3 = tumor extends into major veins or invades adrenal gland or perinephric tissues but not beyond Gerota's fascia; and T4 = tumor invades beyond Gerota's fascia.

### Cell culture

The human RCC cell lines 769-P and ACHN were obtained from Procell Life Science & Technology (Wuhan, Hubei, China). Human embryonic kidney (HEK) 293T cells were obtained from the Cancer Research Center of Xiamen University (Xiamen, Fujian, China). All cells were authenticated by STR profiling according to the cell bank. 769-P cells were cultured in RPMI-1640 medium (PM150110, Procell, Wuhan, China), ACHN cells were cultured in minimum essential medium (MEM, PM150410, Procell, Wuhan, China), and 293T cells were cultured in Dulbecco's modified Eagle's medium (DMEM, HyClone, Palo Alto, CA). All cells were supplemented with 10% fetal bovine serum (FBS, A0500-3011, Cegrogen Biotech, Germany) and 1% penicillin-streptomycin (Invitrogen, Carlsbad, CA, USA) at 37°C in 5% CO_2_.

### Cell transfection

Through transient transfection, we transferred the desired gene into RCC cells for expression. Lentiviral vectors encoding the human shTNFAIP8 and shRNA vector (pSIREN-RetroQ) were donated by the laboratory of Professor Jin Guanghui of the School of Medicine of Xiamen University. We constructed a lentiviral vector encoding the human TNFAIP8 gene, and the empty vector (Plnx-2) was used as a negative control. We used 769-P and ACHN cells in the logarithmic phase for the experiment and then used the transfection reagent (jetPRIME, 114-15, Polyplus-transfection SA, France) to transfect 769-P and ACHN cells. After 24 h and 36 h, we collected cells for the next step in the experiment.

### Wound healing assay

Cells were cultured in a 6-well plate and grown to subconfluence (80%) in medium containing serum after transfection with our target plasmid for 36 h. The cells were washed three times with the medium, and the bottom of the well was scratched with a 200-μl pipette tip. The debris was removed, and the cells were cultured in serum-free media. After incubation for an additional 24 h, cell migration was analyzed in six different microscopic fields and calculated as the percentage of wound healing. Images were captured at 0 and 24 h on a Leica DM4B microscope (Leica, Germany).

### Transwell migration assay

The migration ability of the cells was measured in a 24-well transwell plate (Corning Costar, Tewksbury, MA, USA). A total of 1×10^5^ 769-P and ACHN cells with the indicated treatment in 200 μl of serum-free medium were seeded into the top chamber of each insert, and 600 μl of medium supplemented with 20% FBS was added to the lower chamber. After 24 h of incubation at 37°C, the cells in the top chamber were carefully washed and fixed with 4% paraformaldehyde. The cells in the lower chamber were removed by a cotton swab, stained with 0.5% crystal violet, and air dried. The number of migrated cells was assessed under a microscope in five separate fields of view. Three independent experiments were performed.

### Reverse‑transcription PCR (RT‑PCR) and real‑time quantitative PCR (q‑PCR)

Cells were collected and total RNA was extracted using the TransZol reagent (TransGen Biotech, Beijing, China). RNA (1 μg) was reverse‑transcribed to cDNA using the Fermentas PCR kit (Fermentas, US). Then the PCR products were analyzed by 1.5% agarose gel electrophoresis, and the gels were observed using a gel imaging system (Clinx GenoSens1500, Shanghai, China).

Real-time PCR was performed using TransStart Top Green qPCR SuperMix(TransGen Biotech, Beijing, China) and data collection was performed on a Bio-Rad Biosystems 7500 instrument with SYBR Green (Bio-Rad, Hercules, CA). The mRNAs were quantified by the 2^-△△Ct^ method from their respective Ct values with β-actin as the internal control. All tests were repeated in triplicate. The primers are listed in Table 1.

### Western blotting and antibodies

Whole cell lysates were prepared using RIPA buffer (Sigma-Aldrich, St. Louis, USA) with 1% protease inhibitor cocktail and 1% phenylmethanesulfonyl fluoride (Gold Biotechnology, USA) at 4°C and cleared by centrifugation. Protein concentration was determined by using a Bradford assay (Bio-Rad, Hercules, CA). Equal amount of protein (10-40 µg) were separated by SDS-PAGE and transferred to PVDF membranes (Millipore, Billerica, MA, USA). The membranes were washed and incubated with the following specific primary antibodies at 4°C overnight: rabbit monoclonal antibody against human TNFAIP8 (1:1000; Abcam, ab195810, Suite Cambridge, USA), rabbit polyclonal antibody against E-cadherin (1:5000, Proteintech, 20874-1-AP, Wuhan, Hubei), rabbit polyclonal antibody against N-cadherin (1:2000, Proteintech, 22018-1-AP, Wuhan, Hubei), rabbit polyclonal antibody against ZO-1 (1:500, Proteintech, 21773-1-AP, Wuhan, Hubei), rabbit polyclonal antibody against Vimentin (1:1000, Proteintech, 10366-1-AP, Wuhan, Hubei), and rabbit monoclonal antibody against GAPDH (1:1000; Cell Signaling Technology, #51743, Trask Lane, MA, USA). The next day, the membranes were washed and incubated with horseradish peroxidase (HRP)-conjugated goat anti-mouse IgG and goat anti-rabbit IgG (1:2000; ZSGB-Bio, Beijing, China) for 1 h at room temperature to visualize the immunoreactive bands. Imaging with a BIO-RAD ChemiDoc XRS + detection system (Bio-Rad, Hercules, CA) was performed.

### Histopathological analysis

The tissue was fixed in 4% paraformaldehyde for 24 hours, embedded in paraffin, and baked at 60 °C for 1 hour before sectioning. The sections were subjected to xylene dewaxing, a portion was used for hematoxylin and eosin (HE) staining, and the remaining sections were immersed in 0.01 M citrate buffer and heated to boiling (100 °C) for 5 minutes by autoclaving for dehydration. After antigen retrieval, the sections were blocked with 5% bovine serum albumin (BSA) for 30 minutes at 37 °C and incubated with primary antibodies against TNFAIP8, E-cadherin, N-cadherin, ZO-1, and Vimentin (as previously described) overnight at 4 °C. The next day, the corresponding secondary antibody was incubated at 37 °C for 1 hour. Finally, the expression of TNFAIP8, E-cadherin, N-cadherin, ZO-1, and Vimentin was observed by staining with diaminobenzidine tetrahydrochloride solution (DAB) for 5 minutes. Then, the sections were stained with hematoxylin for 5 minutes, fixed with a neutral resin, and photographed with a microscope (Olympus BX53, Japan). Finally, we used Image-pro plus 6.0 (Media Cybernetics, Inc., Rockville, MD, USA) to analyze the immunohistochemical average optical (AO) signal.

### Immunofluorescence

The day before the experiment, RCC cells were seeded on glass slides and fixed in 4% paraformaldehyde, followed by permeabilization with 0.5% Triton X-100 for 15 min. After washing with phosphate-buffered saline (PBS), the cells were blocked with 10% normal goat serum for 30 min and then incubated overnight at 4 °C with primary antibodies against E-cadherin and Vimentin. The cells were washed with PBS and incubated with Alexa Fluor 488- or 561-conjugated secondary antibodies for 1 h at room temperature; nuclei were stained with 4',6-diamidino-2-phenylindole (Vector Laboratories, Burlingame, CA, USA), and images were captured with a CLSM fluorescence microscope (Zeiss LSM 880+Airyscan, Carl Zeiss AG, Germany).

### Statistical analysis

All statistical analyses were performed by using GraphPad Prism 5 (San Diego, CA) and SPSS 13.0 software (Chicago, IL, USA). Quantitative data are expressed as the mean ± S.D. Significant differences in quantitative data were compared by two-tailed Student's t-test. The significance of the correlation between the expression of indicated proteins and histopathological factors was determined using the Pearson χ2 test. In all samples, P values < 0.05 were considered statistically significant. Statistical significance was determined at *p<0.05, **p<0.01, and ***p<0.001.

## Results

### High TNFAIP8 expression is associated with advanced stage and poor prognosis in ccRCC patients

**Figure 1:**
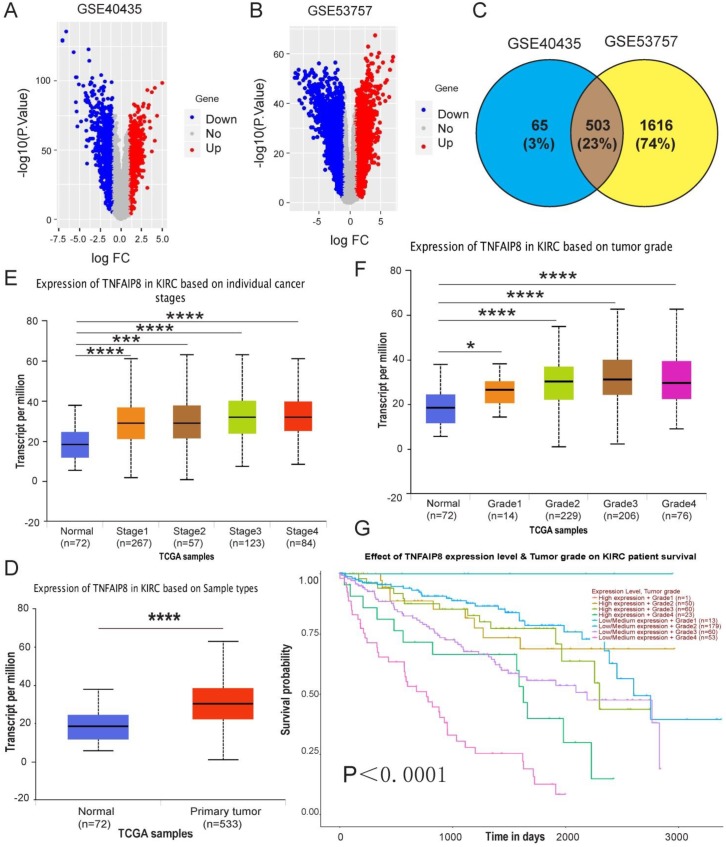
** TNFAIP8 expression in human ccRCC GEO and TCGA-KIRC microarray datasets.** Volcano map depicting TNFAIP8 expression in GEO datasets; red indicates high expression; blue indicates low expression. Both** (A)** GSE40435 and **(B)** GSE53757 confirmed that TNFAIP8 is highly expressed in ccRCC.** (C)** TNFAIP8 was detected at the intersection of the two datasets. **(D)** TNFAIP8 mRNA expression in normal and ccRCC tissues. Histograms showing mRNA upregulation in ccRCC samples relative to that in normal samples (data downloaded from TCGA). Analysis of TNFAIP8 expression in ccRCC at different stages **(E)** and grades **(F)** of TGCA data. **(G)** Kaplan-Meier curve of the effect of TNFAIP8 expression level and tumor grade on survival rate in ccRCC patients. *P < 0.05; ***P < 0.001; ****P<0.0001.

To investigate the role of TNFAIP8 in human ccRCC, we first analyzed available human datasets of ccRCC patients in the Gene Expression Omnibus (GEO) database, and we randomly collected two data sets (GSE40435 and GSE53757) to draw volcano plots. We determined that TNFAIP8 expression was elevated in ccRCC (red region, Figure 1A, Figure 1B). Subsequently, we intersected the data sets and confirmed 503 common genes, and TNFAIP8 was also found in the common area (Figure 1C). Next, we checked the function of TNFAIP8 in ccRCC in the Cancer Genome Atlas (TCGA) database and verified that the relative TNFAIP8 mRNA expression level was significantly increased in tumor tissues compared with that of adjacent tissue (Figure 1D). This information indicated that TNFAIP8 was involved in the tumorigenesis of ccRCC. In addition, we examined TNFAIP8 mRNA levels in tumors of different stages and grades and discovered that TNFAIP8 was significantly higher in T3/T4-stage tumors than in T1/T2-stage tumors (Figure 1E), as well as in T3/T4-grade tumors than in T1/T2-grade tumors (Figure 1F). Finally, we analyzed the relationship between TNFAIP8 and overall survival and showed that differential expression of TNFAIP8 was significantly associated with overall survival (Figure 1G). To determine whether the increased TIPE expression was associated with clinical characteristics, we collected the GSE40435 data and analyzed the gender, age and tumor grade of ccRCC patients and observed that there was no significant correlation with these characteristics (p>0.05) (Supplementary Table 1).

In general, we clarified that TNFAIP8 plays a vital role in the tumorigenesis and development of ccRCC. Our results suggest that TNFAIP8 is highly expressed in ccRCC and that increased expression of TNFAIP8 is associated with advanced stage and poor prognosis in ccRCC patients.

### TNFAIP8 is aberrantly overexpressed in ccRCC tissue samples and ccRCC cell lines

**Figure 2:**
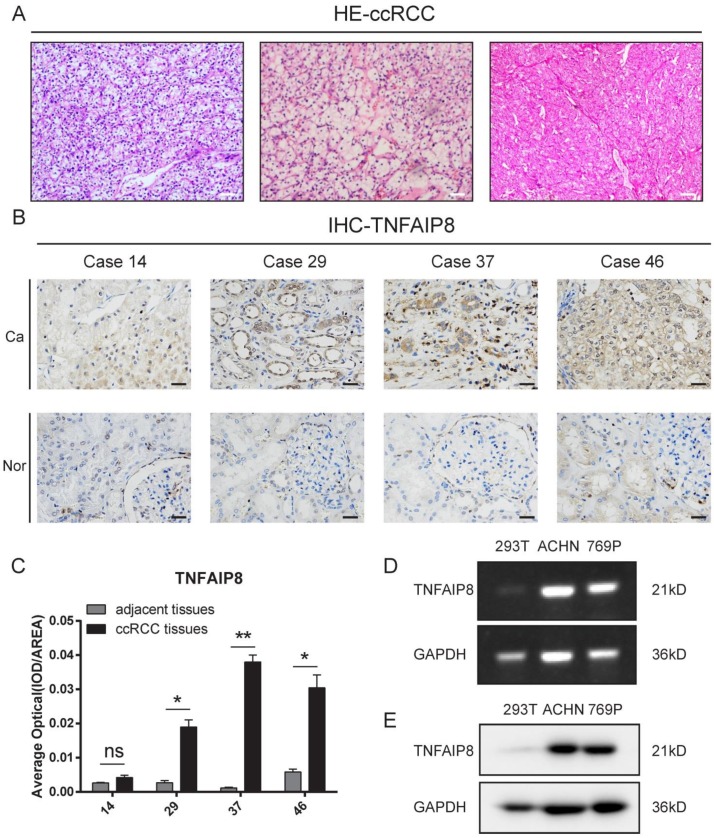
** Detection of TNFAIP8 expression in ccRCC tissue samples and cell lines. (A)** H&E staining of randomly selected ccRCC tissue sections. Scale bar: 200 μm**. (B)** TNFAIP8 expression in randomly paired human ccRCC tissues and matched adjacent tissues analyzed by immunohistochemical staining. Scale bar: 50 μm **(C)** Representative statistical results are shown. ns: no significance; *P < 0.05; **P <0.01. **(D)** The mRNA expression of TNFAIP8 was confirmed by real-time PCR in ccRCC cell lines and compared to that of normal cells.** (E)** Further protein level examination of TNFAIP8 expression in the corresponding cell lines by western blotting.

To determine the role of TNFAIP8 in ccRCC, TNFAIP8 expression was examined in ccRCC and adjacent tissues by hematoxylin and eosin (HE) and immunohistochemistry (IHC) staining. We found that the classic clear cell area of ccRCC and cancer cells was arranged in a bubbling growth pattern, which was rich in blood vessels (Figure 2A). Additionally, TNFAIP8 was highly expressed in the cytoplasm in cancer tissues compared to levels in adjacent noncancerous tissues (Figure 2B). The corresponding statistical analysis found that the expression of TNFAIP8 was significantly increased in ccRCC tissues (Figure 2C). Then, the expression of TNFAIP8 was assessed in a panel of RCC cell lines (769-P and ACHN), with HEK293T cells serving as a control. We found that TNFAIP8 was significantly increased in ccRCC cell lines at the mRNA (Figure 2D) and protein level (Figure 2E) compared to those of 293T cells.

Taken together, these results suggest that TNFAIP8 is aberrantly upregulated in human ccRCC tissues and ccRCC cell lines.

### TNFAIP8 promotes the migration and invasion of ccRCC cells *in vitro*

**Figure 3:**
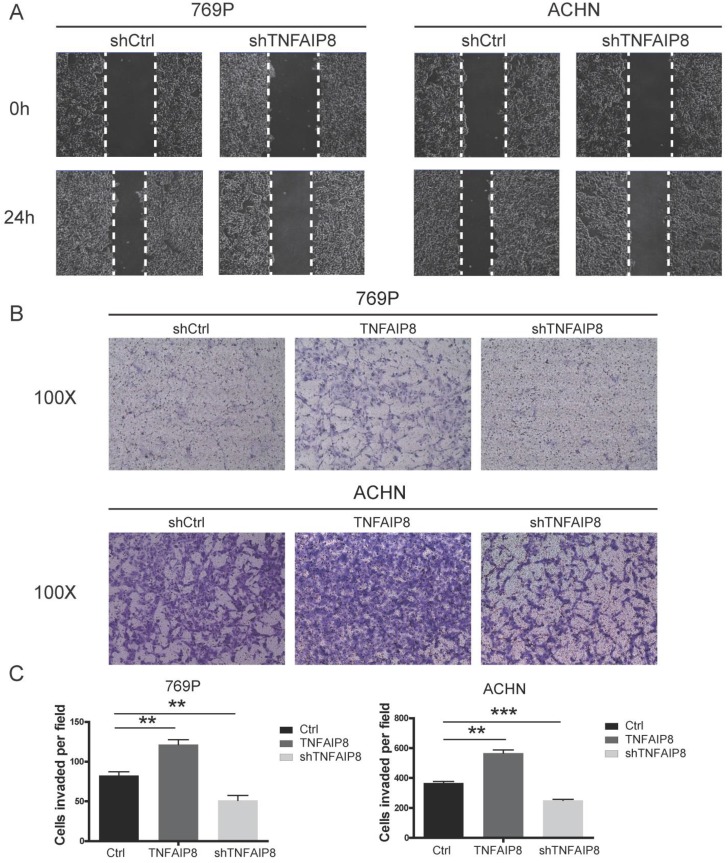
** Changes in the expression of TNFAIP8 affect cell migration.** After transient transfection with shTNFAIP8 or control plasmid for 36 hours, the wound healing ability after scratching was monitored by microscopy. **(A)** Representative images showing at least three independent experiments in the ccRCC cell lines 769-P and ACHN. **(B)** Using a transwell assay, cell migration was significantly enhanced after transfection with TNFAIP8 in 769-P and ACHN cells but decreased after transfection with shTNFAIP8. Quantitative analysis of three independent experiments is shown in** (C)**; ** P <0.01, *** P < 0.001.

To confirm the potential cellular functions of TNFAIP8 in ccRCC, we constructed TNFAIP8 knockdown and overexpression lines in 769-P and ACHN cells by transient transfection. The scratch assay demonstrated that cell migratory ability was significantly decreased in the TNFAIP8-shRNA group compared with that of the control-shRNA group (Figure 3A). Transwell assays further showed that the depletion of TNFAIP8 dramatically attenuated migration compared with that of control cells. Consistent with the knockdown results, when overexpressing TNFAIP8, the cell migration ability was significantly increased compared to those of the control and knockdown groups (Figure 3B). Furthermore, we performed statistical analysis after cell counting. There was a statistically significant difference between overexpression or knockdown and the control group (Figure 3C). Similarly, we performed invasion experiments with 769-P cells and found that overexpressing TNFAIP8 promoted cell invasion, while knockdown of TNFAIP8 reduced invasion (Supplementary Figure 1A, B).

These results demonstrate that TNFAIP8 expression levels are positively correlated with ccRCC cell migration and that TNFAIP8 promotes the migration and invasion of ccRCC cells.

### TNFAIP8 regulates epithelial‑mesenchymal transition (EMT)-associated molecule expression

The epithelial‑mesenchymal transition (EMT) has been reported to play a crucial role in migration and further cause cancer metastasis 14-16. To determine how TNFAIP8 controls ccRCC migration, the possible effects of TNFAIP8 on the EMT were assessed. We examined the expression of E-cadherin and zonula occludens-1 (ZO-1) as epithelial markers and N-cadherin and vimentin as mesenchymal markers.

**Figure 4:**
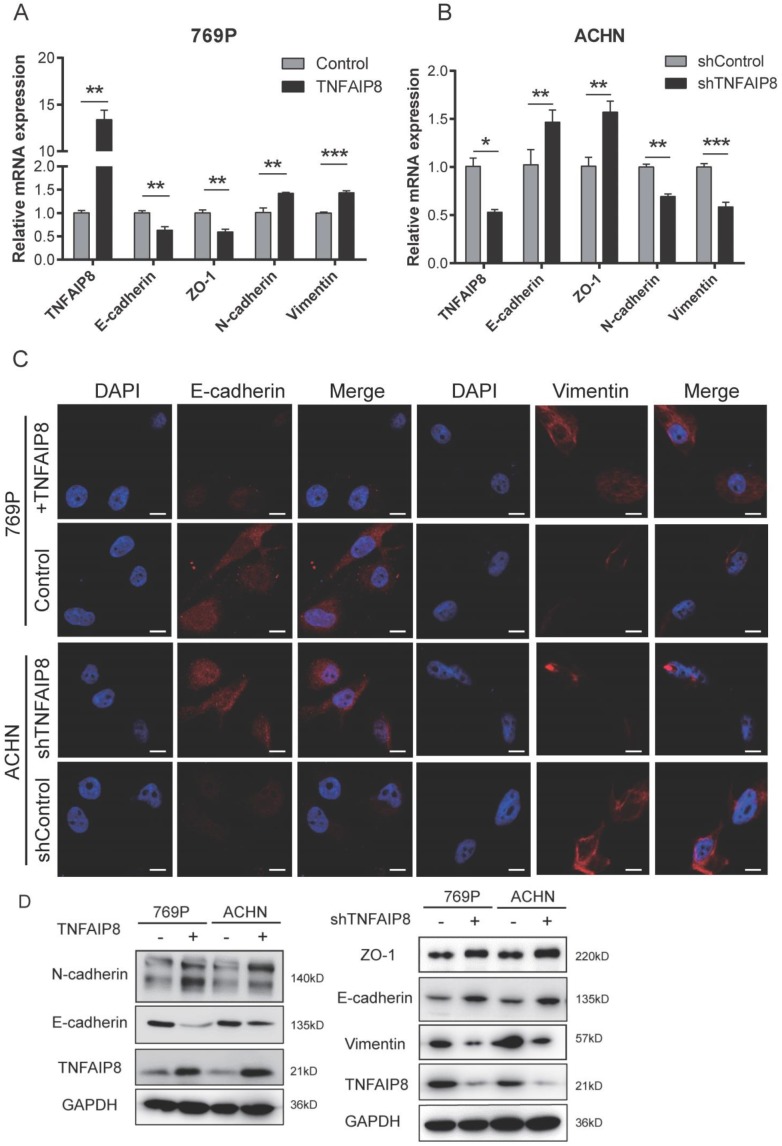
** Alteration in TNFAIP8 expression regulates expression of EMT-related molecules in ccRCC cells.** TNFAIP8, E-cadherin, zonula occludens (ZO)-1, N-cadherin, and vimentin expression was evaluated by real-time PCR in 769-P cells **(A)** and ACHN cells **(B)**. **(C)** Immunofluorescence analysis of E-cadherin and vimentin expression in 769-P and ACHN cells. **(D)** Expression of the abovementioned EMT markers after abnormal expression of TNFAIP8 in 769-P and ACHN cells (TNFAIP8, Con; shTNFAIP8, shCon), as determined by western blotting. The data were obtained from three independent experiments. Scale bar: 100 μm, *P < 0.05; **P < 0.01; ***P < 0.001.

We know that TGF-β is the main inducer of the EMT, and so when TGF-β is increased, EMT induction is effective 17-20. We first tested certain molecules correlated with TGF-β/EMT signaling and found that when TNFAIP8 was increased, TGFβ, ZEB1, ZEB2, Twist, and Slug also increased, while Snail decreased (Supplementary Figure 1C); converse results were found when TNFAIP8 was decreased (Supplementary Figure 1D). These results indicate that the EMT is induced and activated when TNFAIP8 is elevated. Then, we overexpressed TNFAIP8 in 769-P cells and knocked down TNFAIP8 in ACHN cells. We confirmed that when TNFAIP8 was increased, the epithelial markers E-cadherin and ZO-1 were downregulated, whereas the mesenchymal markers N-cadherin and vimentin were upregulated. These results were confirmed by real-time PCR (Figure 4A, B) and immunofluorescence (Figure 4C) analyses. We overexpressed TNFAIP8 in both 769-P and ACHN cells and found that TNFAIP8 induced a significant increase in N-cadherin expression, whereas E-cadherin protein levels were reduced. We further knocked down the expression of TNFAIP8 at the cellular level and demonstrated that the expression of E-cadherin and ZO-1 were significantly increased, but vimentin was decreased (Figure 4D).

These results indicate that overexpression of TNFAIP8 induces the EMT process while reducing TNFAIP8 expression inhibits EMT development, providing evidence that TNFAIP8 regulates the EMT in ccRCC cells, thereby promoting migration and metastasis in ccRCC.

### TNFAIP8 regulates EMT processes in clinical ccRCC samples

**Figure 5:**
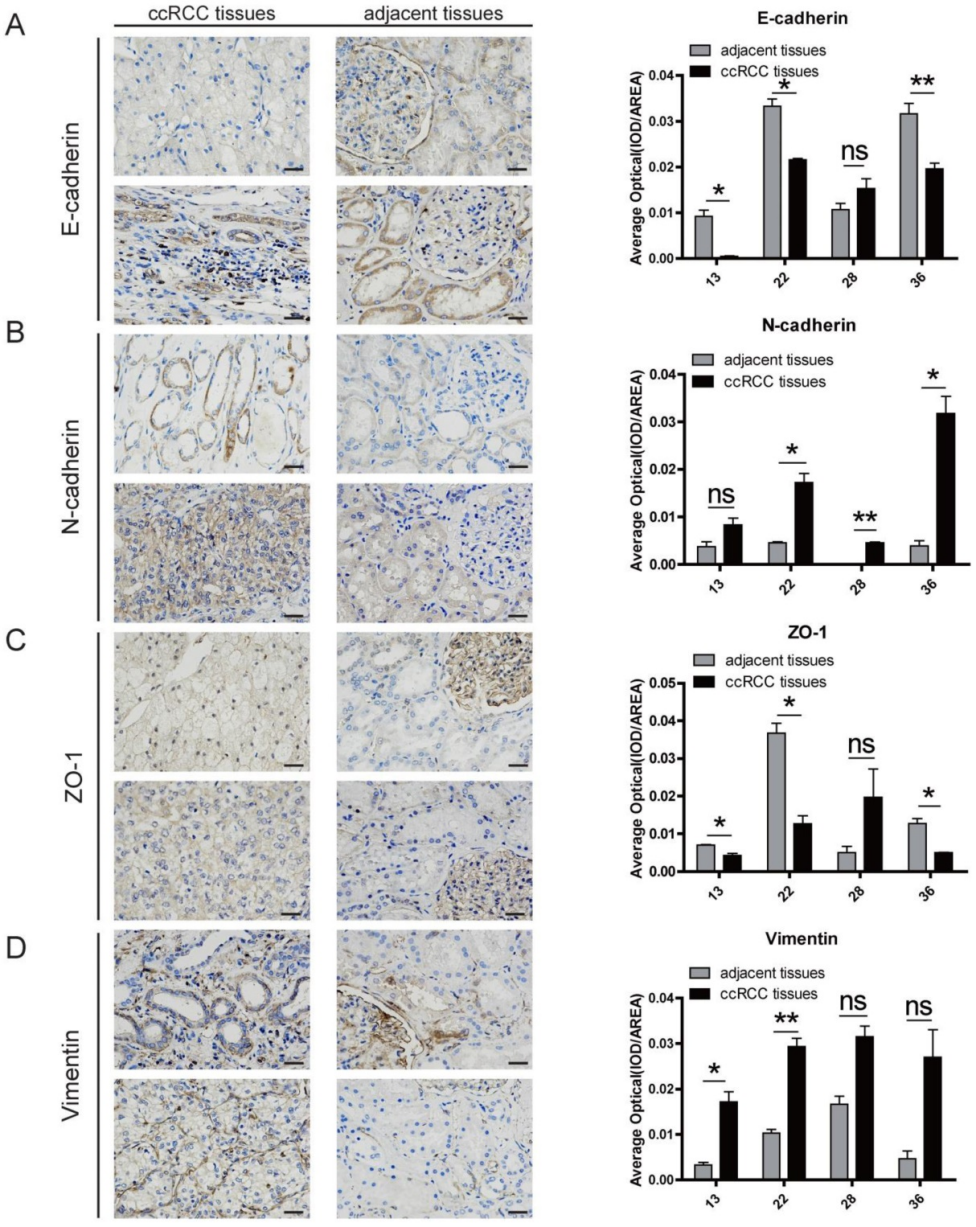
** TNFAIP8 regulates EMT processes in clinical ccRCC samples.** Immunohistochemical staining analysis of the levels of **(A)** E-cadherin, **(B)** N-cadherin,** (C)** zonula occludens (ZO)-1, and **(D)** vimentin in ccRCC tissues and adjacent noncancerous tissues. Neighboring statistical analysis of the abovementioned EMT marker quantification when TNFAIP8 was highly expressed. Scale bar: 50 μm, ns: no significance; *P < 0.05; **P < 0.01.

Since we confirmed the regulatory role of TNFAIP8 in cells, we also verified whether the same effect existed in clinical samples. We examined clinical samples with a high expression of TNFAIP8 to determine whether the EMT is consistent with the findings in ccRCC cell lines. We randomly selected five clinical samples and removed one sample that did not express TNFAIP8 and found that the relative expression levels of E-cadherin and ZO-1 in the ccRCC group were decreased compared with that of the adjacent normal groups (Figure 5A, C), but N-cadherin and vimentin were increased in the ccRCC group (Figure 5B, D). In addition, we compared the results of ccRCC tissues with adjacent noncancerous tissues and found that there was a significant difference between the two when the expression of TNFAIP8 increased (Figure 5A-D).

These results suggest that TNFAIP8 also regulates EMT processes in clinical ccRCC samples by affecting the expression levels of associated factors.

## Discussion

ccRCC is characterized by high invasiveness, high mortality, and resistance to conventional treatment regimens including chemotherapy and radiation^21^, ^22^. In addition, most ccRCC patients are already in the advanced stage at diagnosis, and there are currently no biomarkers for early diagnosis or treatment of ccRCC 23, resulting in a relatively poor prognosis for ccRCC 24. Therefore, it is necessary to understand the mechanism behind the initiation of metastasis in ccRCC and to find effective therapeutic targets for treating metastatic ccRCC 21.

The EMT plays an important role in RCC development, invasion, and metastasis formation 25. Studies have also shown that the EMT is associated with an increased risk of recurrence and the worst overall survival (OS) in patients with RCC 26. In RCC, the EMT can be induced by a variety of factors, such as TNF-α 27, deletion of VHL 28, and dysregulation of miRNAs 29, 30. However, the molecular mechanisms regulating EMT processes in ccRCC cells are still elusive, and so finding new EMT-associated targets in the development, metastasis, and treatment of RCC remains encouraging.

TNFAIP8 is the first discovered member of the TNFAIP8 protein family and plays a major role in anti-apoptosis and carcinogenesis processes 31. Recent studies have shown that the expression level of TNFAIP8 is associated with tumor stage and lymph node metastasis (TNM) in esophageal squamous cell carcinoma 32, and similar phenomena have been observed in pancreatic cancer 33, gastric adenocarcinoma 34, breast cancer 35, and endometrial cancer 36. In addition, TNFAIP8 is also a risk factor for non-Hodgkin's lymphoma 37; high levels of TNFAIP8 expression are also associated with more aggressive epithelial ovarian cancer 38 and nonsmall cell lung cancer 39. The expression of TNFAIP8 is also increased in diabetic kidneys. At high glucose levels, the expression of TNFAIP8 in mesangial cells is upregulated, and the proliferation of mesangial cells is enhanced 40. However, there are few studies on TNFAIP8 in ccRCC.

In this study, we linked TNFAIP8 to the EMT and analyzed its role in ccRCC. We first found through bioinformatics analysis that the TNFAIP8 expression level was significantly increased in ccRCC, was positively correlated with tumor stage and grade and was associated with poor prognosis in patients with advanced ccRCC. Subsequently, we demonstrated high expression of TNFAIP8 in clinical ccRCC samples and tumor cells. In addition, we found that TNFAIP8 promotes the migration and invasion of ccRCC cells, thereby affecting the metastasis of ccRCC. Finally, we validated the relationship between TNFAIP8 and EMT-specific epithelial genes (such as E-cadherin and ZO-1) and mesenchymal genes (such as N-cadherin and Vimentin) by qPCR, WB, IHC, and IF, confirming that TNFAIP8 regulates EMT-related molecule expression and ultimately affects the EMT process in ccRCC. Taken together, these findings indicate that TNFAIP8 exerts its biological function in ccRCC by regulating the EMT.

In conclusion, this research demonstrates for the first time that TNFAIP8 plays a role in ccRCC, which subsequently leads to metastasis of ccRCC by regulating the EMT, suggesting that TNFAIP8 may be a potential therapeutic target for treating advanced ccRCC. Although some new insights into the metastasis of ccRCC have been provided by our study, the mechanism by which TNFAIP8 is upregulated in ccRCC and its pathways for regulating the EMT need to be elucidated in future studies.

## Supplementary Material

Supplementary figure and table.

## Figures and Tables

**Table 1:** Primer sequences for RT PCR and q-PCR.

**RT‑PCR primer sequence**		
**Primers**	**Forward/Reverse**	**5'-3'**
**GAPDH**	F	CAAGGCTGTGGGCAAGGTCATC
	R	GGAGTGGGTGTCGCTGTTGAAG
**TNFAIP8**	F	TGTCCAAATCCATCGCCACCAC
	R	CCGTCCATGTGACTTGGCAGTG
**q-PCR primer sequence**		
**Primers**	**Forward/Reverse**	**5'-3'**
**Actin**	F	AGCGAGCATCCCCCAAAGTT
	R	GGGCACGAAGGCTCATCATT
**TGF-β**	F	AAGGACCTCGGCTGGAAGTGG
	R	GGACCTTGCTGTACTGCGTGTC
**E-cadherin**	F	CGCCATCGCTTACACCATCCTC
	R	CTCTCTCGGTCCAGCCCAGTG
**N-cadherin**	F	TGCCATCATTGCCATCCTGCTC
	R	CCCGGCGTTTCATCCATACCAC
**Vimentin**	F	ACCAGCCGCAGCCTCTACG
	R	AGCGAGAAGTCCACCGAGTCC
**ZO-1**	F	AGGAGGTAGAACGAGGCATCATCC
	R	TCTCCAGAAGTCAGCACGGTCTC
