# Supplementary material for: TNFAIP8 promotes the migration of clear cell renal cell carcinoma by regulating the EMT
Source: J Cancer. 2020 Mar 4;11(10):3061–71. doi: 10.7150/jca.40191 (PMC7086265; doi:10.7150/jca.40191)
Supplement: Supplementary file 1 — Supplementary figure and table. [file jcav11p3061s1.pdf]

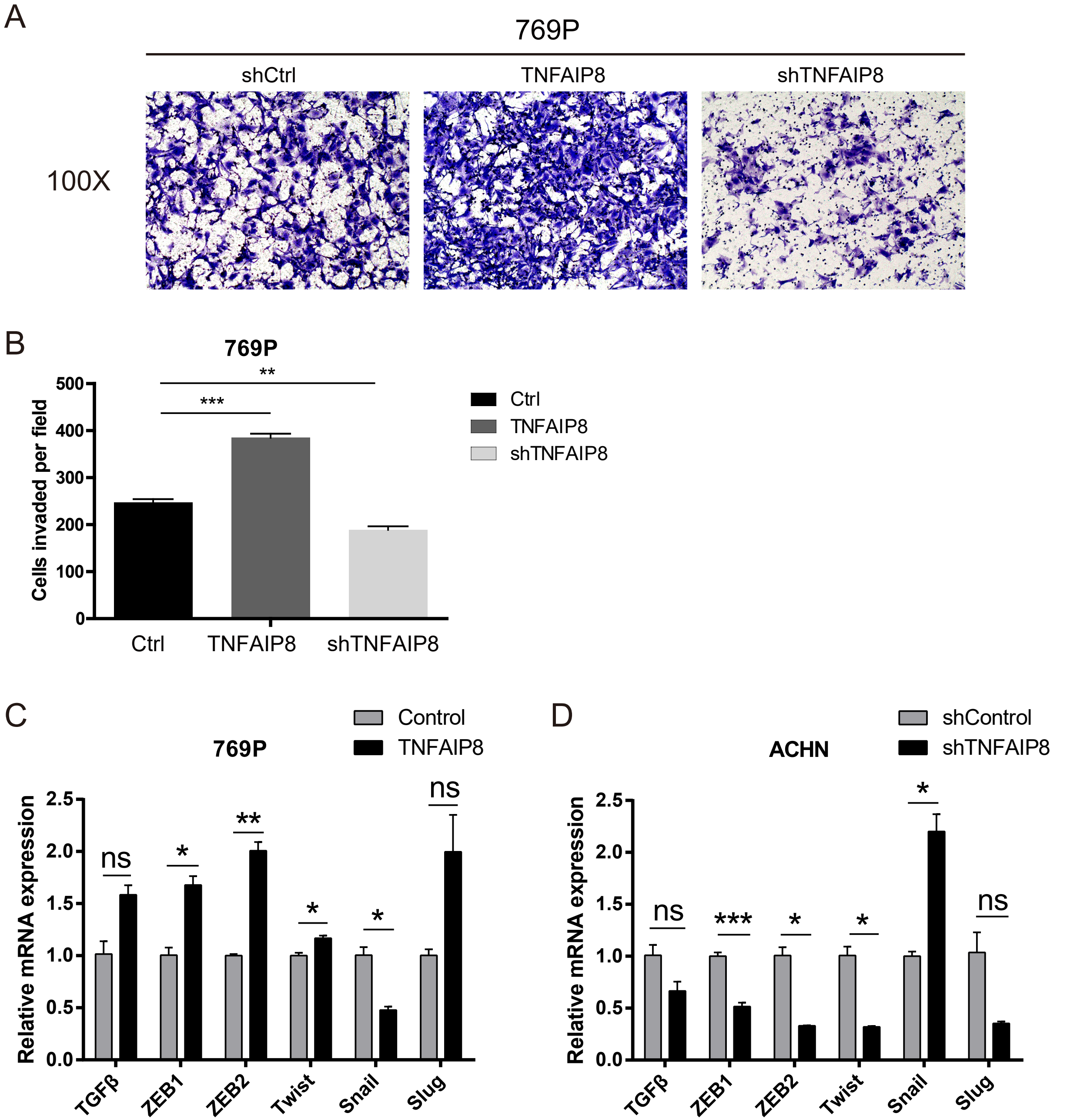
A
769P
shCtrl
TNFAIP8
shTNFAIP8
100X
B
769P
Cells invaded per field
500
400
300
200
100
0
***
**
Ctrl
TNFAIP8
shTNFAIP8
Ctrl
TNFAIP8
shTNFAIP8
C
769P
Control
TNFAIP8
Relative mRNA expression
2.5
2.0
1.5
1.0
0.5
0.0
ns
*
**
*
*
ns
TGFβ
ZEB1
ZEB2
Twist
Snail
Slug
D
ACHN
shControl
shTNFAIP8
Relative mRNA expression
2.5
2.0
1.5
1.0
0.5
0.0
ns
***
*
*
*
ns
TGFβ
ZEB1
ZEB2
Twist
Snail
Slug

Supplementary Table 1. Distribution of tumor necrosis factor-α induced protein 8 (TNFAIP8) expression in clear cell renal cell carcinoma patients according to clinicopathological characteristics.

**Characteristics**	**TNFAIP8**		χ^2	**P value**
	Positive	Negative		
**Gender**				
Male	52	7	0.7753	0.3786
Female	40	2		
**Age**				
≥64Y	51	3	0.8438	0.3583
<64Y	41	6		
**Grade**				
Grade I	19	3	0.8937	0.6397
Grade II	43	4		
Grade III	22	2		
Grade IV	8	0		
